# Longitudinal Trajectories of Global and Domain-Specific Cognition After Stroke Using the Oxford Cognitive Screen

**DOI:** 10.1161/STROKEAHA.125.054555

**Published:** 2026-05-19

**Authors:** Elise Milosevich, Andrea Kusec, Sarah T. Pendlebury, Nele Demeyere

**Affiliations:** Investigative Medicine Division, Radcliffe Department of Medicine (E.M.), University of Oxford, United Kingdom.; Nuffield Department of Clinical Neurosciences (A.K., N.D.), University of Oxford, United Kingdom.; Nuffield Department of Clinical Neurosciences, Wolfson Centre for Prevention of Stroke and Dementia (S.T.P.), University of Oxford, United Kingdom.; NIHR Oxford Biomedical Research Centre and Departments of General Medicine and Geratology, John Radcliffe Hospital, Oxford, United Kingdom (S.T.P.).

**Keywords:** cognition, cognitive dysfunction, follow-up studies, long-term care, stroke

## Abstract

**BACKGROUND:**

Cognitive impairment is common after stroke and linked to poor outcomes, yet long-term recovery or decline, particularly across specific cognitive domains, remains unclear. Most studies use brief global screeners with short follow-up, limiting insight into recovery patterns. This study aimed to characterize domain-specific cognitive trajectories over ≥2 years poststroke and identify predictors of persistent impairment.

**METHODS:**

Participants were recruited at a regional acute stroke unit (John Radcliffe Hospital, Oxford, United Kingdom; 2012–2019) and assessed acutely, at 6 months, and ≥2 years poststroke. The Oxford Cognitive Screen was administered at all timepoints. Global impairment severity was quantified by the proportion of Oxford Cognitive Screen subtasks impaired. Logistic mixed-effects models examined longitudinal change and predictors of domain-specific impairments (language, memory, attention, executive function, and number processing). Latent class growth analysis identified distinct cognitive trajectories. Models were adjusted for acute cognitive impairment severity and time.

**RESULTS:**

Of 866 patients assessed acutely, 105 were followed up at ≥2 years (98 with complete Oxford Cognitive Screen data; median, 4.1 [interquartile range, 3.3] years; mean age, 69 years, 41% female). Cognitive impairment severity improved substantially by 6 months (β=−0.11; *P*<0.001) and further long-term (β=−0.15; *P*<0.001). Acute impairment severity strongly predicted long-term outcomes (β=0.50; *P*<0.001), while demographic and vascular factors explained minimal variance. Latent class growth analysis identified 4 overall trajectories: no or mild acute impairment with stability (47.6%), moderate-improving (32.3%), large improvement (15.2%), and decline (4.8%). Domain-specific improvements were greatest in memory (odds ratio, 16.40 [95% CI, 5.52–48.7]) and language (odds ratio, 8.17 [95% CI, 3.17–21.1]), more limited in attention (odds ratio, 5.41 [95% CI, 2.52–11.6]) and executive function (odds ratio, 4.14 [95% CI, 1.96–8.75]). Domain models revealed additional classes of persistent or delayed recovery, particularly in executive function and attention.

**CONCLUSIONS:**

Cognitive recovery is most pronounced within 6 months and continues across domains though executive dysfunction often persists. Acute impairment severity best predicted long-term outcomes, while vascular and demographic factors were less informative. Distinct trajectory classes highlight the need for individualized, long-term cognitive monitoring to guide rehabilitation and prognostication. These findings underscore the importance of long-term cognitive follow-up in stroke care and provide empirical benchmarks for recovery across domains.

Cognitive impairment is common following stroke^1,2^ and frequently contributes to poor functional outcomes,^3,4^ increased dependency,^5^ and reduced quality of life.^4^ While many individuals experience some degree of cognitive recovery, others may show persistent impairment or delayed decline.^3,6^ However, the long-term trajectories of change across different cognitive domains remain poorly understood.^7^ This is partly due to cognitive deficits often being overlooked beyond the immediate postacute period unless dementia develops, which is not an inevitable outcome.^6,8,9^

**See related article, p 2163**

Most longitudinal studies of poststroke cognition have assessed performance at only 1 or 2 timepoints within the first year^10^ often using brief global screening tools or undifferentiated assessments.^6^ While these tools offer a general overview, they lack the sensitivity to detect domain-specific patterns of impairment and recovery that are common following stroke.^11^ Furthermore, cognition is frequently conceptualized dichotomously, either impaired or unimpaired, masking within-person variability and limiting insight into the heterogeneity of outcomes. Even large-scale efforts to map long-term trajectories, such as the recent pooled analysis of over 20 000 individuals across 14 cohorts,^12^ found consistent evidence of acute and accelerated poststroke cognitive decline but were constrained by variability in cognitive measures and limited resolution of domain-specific change. Consequently, it remains unclear whether a change in specific cognitive domains may occur in later years, as observed in scarce long-term (10-year) follow-up studies.^8,13^

Determining which cognitive domains are most susceptible to long-term decline, and which early impairments persist, resolve, or fluctuate, is essential for improving prognostic accuracy and informing long-term care planning^14^ and extending the care pathway to cognitive monitoring.^15^ While several risk factors for poststroke cognitive impairment have been identified, such as age, stroke severity, education level, and vascular comorbidities,^9,16^ a recent systematic review highlighted the most predictive factor to be baseline cognition.^17^ However, few studies have investigated how this relates to domain-specific cognitive trajectories over extended periods.

To address the critical knowledge gap around long-term domain-specific cognitive trajectories after stroke, this study aimed to delineate long-term trajectories of global and domain-specific cognitive function following stroke and to examine how early cognitive impairments, clinical variables, and demographic factors relate to different patterns of cognitive recovery or decline.

## Methods

### Data Availability

Deidentified study data are freely available for public research use via an application to https://www.dementiasplatform.uk/.

### Participants

Participants were initially recruited through the Oxford Cognitive Screening program based within the regional acute stroke unit at the John Radcliffe Hospital, United Kingdom, from 2012 to 2019 (National Research Ethics Committee [United Kingdom] approval references 14/LO/0648 and 18/SC/0550). Participants were included if they had a confirmed stroke diagnosis, were aged ≥18 years, and had sufficient English language comprehension to understand assessment instructions. Patients were excluded if they were unable to provide written or witnessed informed consent or unable to concentrate for 20 minutes as judged by the multidisciplinary team. A total of 866 stroke patients were recruited and received cognitive screening acutely (≤2 weeks post-admission; timepoint 1 [T1]), 430 (49.7%) of whom completed 6-month follow-up (T2). Following this, 208 individuals consented to being recontacted for potential inclusion in further research and were invited to participate in the OX-CHRONIC (Oxford Chronic Stroke Study) long-term follow-up study (UK REC Ref: 19/SC/0520).

**Figure 1.**
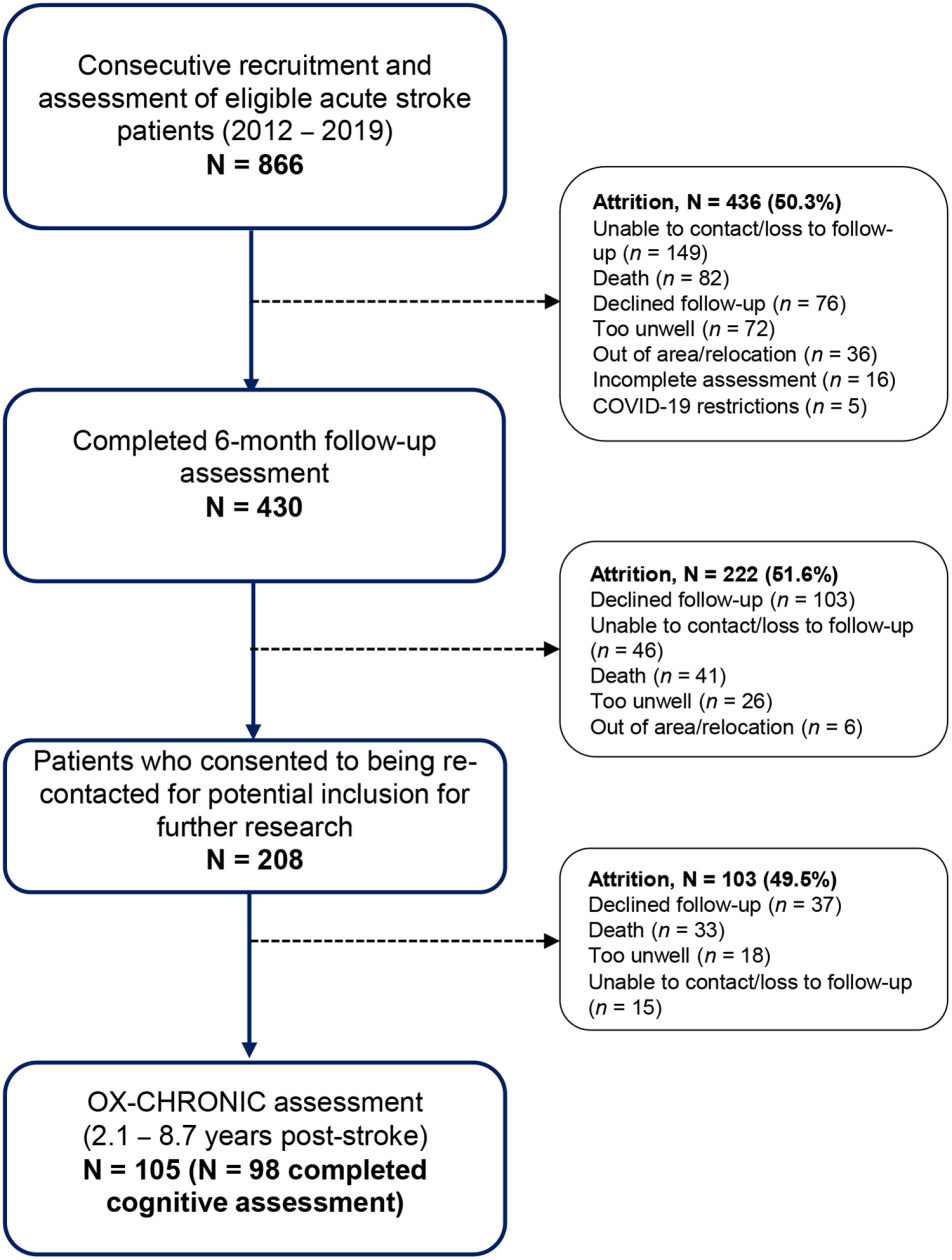
**Flowchart of participant recruitment and attrition across all assessment timepoints at acute, 6 months, and ≥2 years poststroke (chronic).** OX-CHRONIC indicates Oxford Chronic Stroke Study.

A total of 105 participants were enrolled in OX-CHRONIC, 98 of whom completed cognitive assessments ≥2 years poststroke (T3). Detailed recruitment and attrition across all timepoints are summarized in Figure 1, with demographic information of those retained versus those lost to attrition in Figure S1. Patient demographics, comorbidities, vascular risk factors, stroke characteristics, neuroimaging, and laboratory data were collected on admission or retrospectively through electronic medical records. Further information outlining the OX-CHRONIC study design was published previously^18,19^ together with detailed data on attrition at 6 months^2^ and beyond (OX-CHRONIC).^19^

### Cognitive Assessment

At all 3 timepoints, each participant received domain-specific cognitive screening with the Oxford Cognitive Screen (OCS).^20^ The 12 OCS subtest scores were categorized into 5 cognitive domains: language (picture naming, semantic understanding, and sentence reading), attention (egocentric and sustained spatial attention, and allocentric attention), executive function (trail-making), memory (orientation, verbal recall and recognition, and episodic recognition), and number processing (calculations and number writing). Subtests were binarized into impaired or unimpaired based on published normative scores for each subtest.^18,20^ A domain impairment was characterized as at least 1 impaired subtest in that domain, as the number of subtests ranges from 1 to 3 across domains. To measure the severity of cognitive impairment continuously, we utilized the proportion of OCS subtests impaired divided by the total number of subtests completed in analyses to provide a more granular interpretation of the data.

Due to COVID-19 disruption, the third timepoint (OX-CHRONIC) assessment was performed remotely through videoconferencing or telephone calls. A testing pack was mailed to the participant, and the assessor provided instructions throughout the call, asking for verbal (or nonverbal, for example, tapping phone, completing paper, and pencil tasks in the workbook) responses to tasks. This remote testing methodology using the OCS has since been validated,^21^ and remote neuropsychological assessment more generally has been found to be reliable.^22^ Due to the remote nature of assessment, the OCS praxis domain (gesture imitation) could not be tested, and therefore, only 5 cognitive domains were assessed within those assessed at ≥2 years poststroke: language, attention, executive function, memory, and number processing. The Montreal Cognitive Assessment^23^ was used to assess general cognitive status at long-term follow-up (T3) to increase comparability of cognitive function with other studies.

### Statistical Analysis

All analyses were conducted using R, version 4.4.0. Descriptive statistics were used to summarize sample demographics and clinical characteristics, and to report prevalence rates of global and domain-specific impairments at each timepoint (acute, 6 months, and chronic follow-up).

To examine longitudinal trajectories of cognitive function over time, a 3-stage analytic approach was applied across global and domain-specific outcomes of the 105 participants and 98 with complete OCS data at T3. The 7 with missing T3 OCS data were still retained in analyses as models accounted for unbalanced data using maximum likelihood estimation.

First, global cognitive function was analyzed using linear mixed-effects models, with the proportion of OCS subtasks impaired (severity of impairment) as the outcome. Fixed effects included timepoint (acute, 6 months, and chronic), and random intercepts were included to account for within-subject clustering. Model 1 included time only; model 2 added the proportion of acute OCS impairments; and model 3 further adjusted for age, sex, education, lesion hemisphere, acute National Institutes of Health Stroke Scale (NIHSS) score, atrial fibrillation, hypertension, diabetes, smoking status, stroke recurrence, and days poststroke. Models were fit using maximum likelihood estimation, with cases containing missing data excluded. Model performance was evaluated using marginal and conditional R^2^, intraclass correlation coefficients, and fit information criteria (Akaike information criterion [AIC] and Bayesian information criterion [BIC]). Pairwise comparisons between timepoints were assessed using Tukey-adjusted post hoc tests.

Second, domain-specific impairment was modeled using mixed-effects logistic regression. For each domain (language, memory, attention, executive function, and number processing), impairment was defined as at least 1 subtask impaired (0=at least 1 subtask impaired and 1=unimpaired). Models were adjusted for time and baseline impairment status, with random intercepts per participant; no additional covariate adjustment was applied. Model performance was reported using marginal and conditional R^2^ and intraclass correlation coefficients. Odds ratios (ORs) and 95% CIs were calculated for all fixed effects, and Wald z-tests were used to evaluate statistical significance, with ORs signifying the likelihood of cognitive recovery.

Third, to identify distinct subgroups of cognitive trajectories over time, latent class growth analysis was conducted using the *lcmm* package.^24^ Separate models were fitted for global cognition and each cognitive domain. For global cognition and the domains of language, memory, attention, and number processing, the outcome was the proportion of OCS subtasks impaired at each timepoint to allow for greater sensitivity in detecting classes. For executive function, raw mixed trail-making task scores were used. Models specifying 1 to 5 latent classes were fitted, with time as a fixed effect. Model fit was evaluated using standard information criteria, including the AIC (a measure of model fit that penalizes for a greater number of parameters), BIC (a fit measure that penalizes the number of parameters more heavily), sample-adjusted BIC (SABIC), and log likelihood. Final model selection was guided by a combination of statistical fit, parsimony, and clinical interpretability of the identified trajectories, with entropy (H; measure of class separation) values ≥0.80 considered indicative of acceptable classification certainty. For each selected model, trajectory class membership was compared across demographic (age, sex, and education), clinical (NIHSS score, stroke type, recurrence, and lesion hemisphere), and neuroimaging variables (lesion volume, white matter hyperintensities, and cortical atrophy), as well as acute and chronic cognitive functioning (number of acute domains impaired and Montreal Cognitive Assessment score at follow-up). We additionally explored between-group statistical comparisons of global severity of cognitive impairment subgroups using 1-way ANOVAs, with due caution in interpretation due to potentially small group sizes and limited power. This study was reported in accordance with the STROBE guidelines (Strengthening the Reporting of Observational Studies in Epidemiology) for observational studies (Supplemental Material).

## Results

The 105 patients in OX-CHRONIC at the time of stroke had a mean age of 68.9 (SD, 13.0) years, and 40.9% were female, with a mean of 13.9 years of education (SD, 3.7) and a median acute NIHSS score of 7 (interquartile range, 5). Further demographics and acute clinical characteristics of the cohort are outlined in Table 1. Attrition from acute to long-term follow-up was ≈50% between each recruitment stage, primarily due to participants declining further follow-up or death (Figure 1).

**Table 1.**
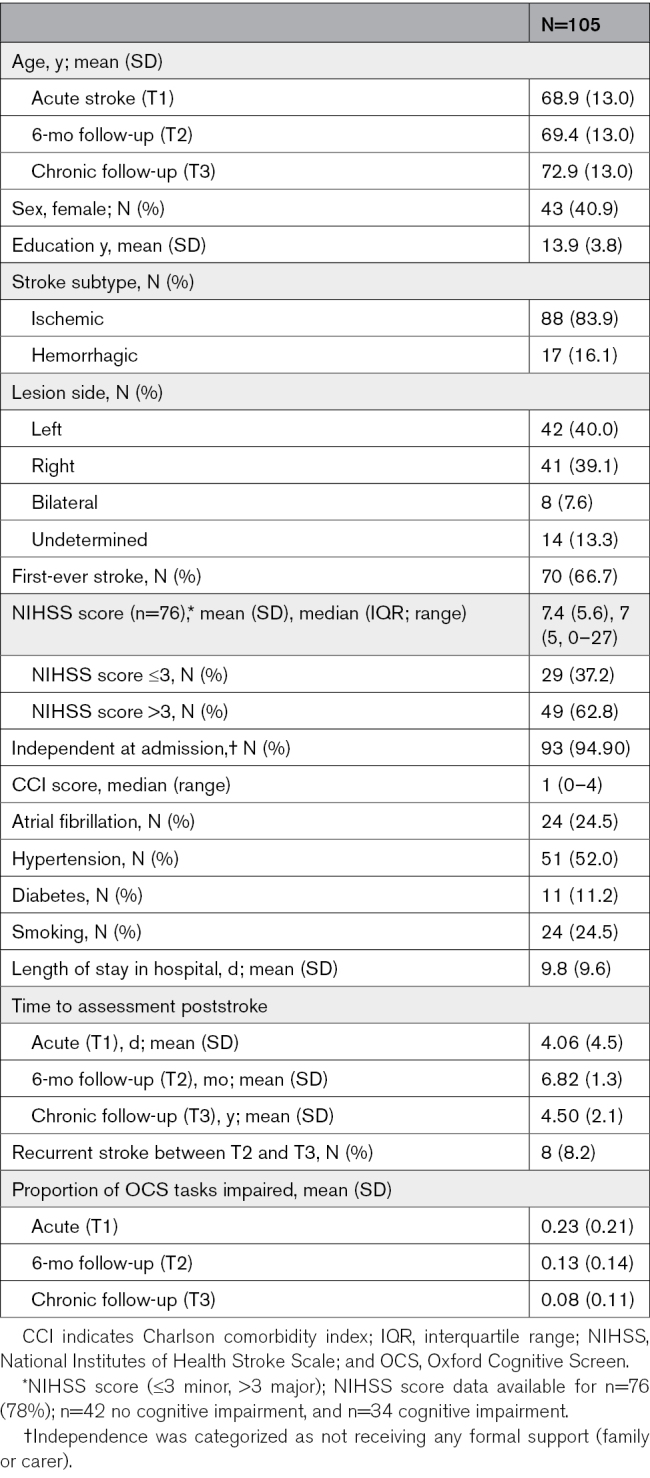
Chronic Stroke Cohort Baseline Demographics and Clinical Characteristics

Full descriptive statistics of acute demographic, clinical, and cognitive characteristics across global cognitive trajectory subgroups are presented in Table S1. OCS cognitive assessments were completed at long-term follow-up (T3) in 98 patients at a mean of 4.5 (SD, 2.1) years after stroke (range, 2.0–9.4 years). Details regarding the prevalence of domain-specific impairments at long-term follow-up are outlined in Table S2. Prevalence of acute and chronic cognitive impairment status stratified by lesion hemisphere is presented in Table S3. At acute (T1), language (χ^2^=10.3; *P*<0.05), and memory (χ^2^=10.2; *P*<0.05) impairments were more common in those with left hemisphere lesions, and attention impairments were more common in those with right hemisphere lesions (χ^2^=12.2; *P*<0.01). There were no differences in cognitive impairment status by lesion hemisphere at long-term follow-up (T3).

### Predicting Overall Severity of Cognitive Impairment Over Time

Mixed-effects models examining overall cognitive function, indexed by the proportion of OCS subtasks impaired, showed significant recovery over time (model 1: β=−0.11 at 6 months and −0.15 at ≥2 years; *P*<0.001). In real terms, the sample on average went from having 23% of tasks impaired acutely (≈3 of 12 OCS tasks impaired) to, on average, having 12% of tasks impaired at 6 months (1–2 OCS tasks impaired) and 8% at chronic follow-up (1 task impaired).

The inclusion of acute impairment status (model 2) explained over half of the variance (R^2^=0.52), with higher acute burden predicting greater persistent deficits (β=0.50; *P*<0.001). A fully adjusted model (model 3) confirmed the predictive role of acute impairment but found no added explanatory value from demographic or clinical covariates, with covariates collectively contributing to only 3% of the variance (model 3: R^2^=0.55). Random-effect variance dropped to zero once acute status was included, indicating minimal residual between-subject heterogeneity. Full model results are presented in Table 2.

**Table 2.**
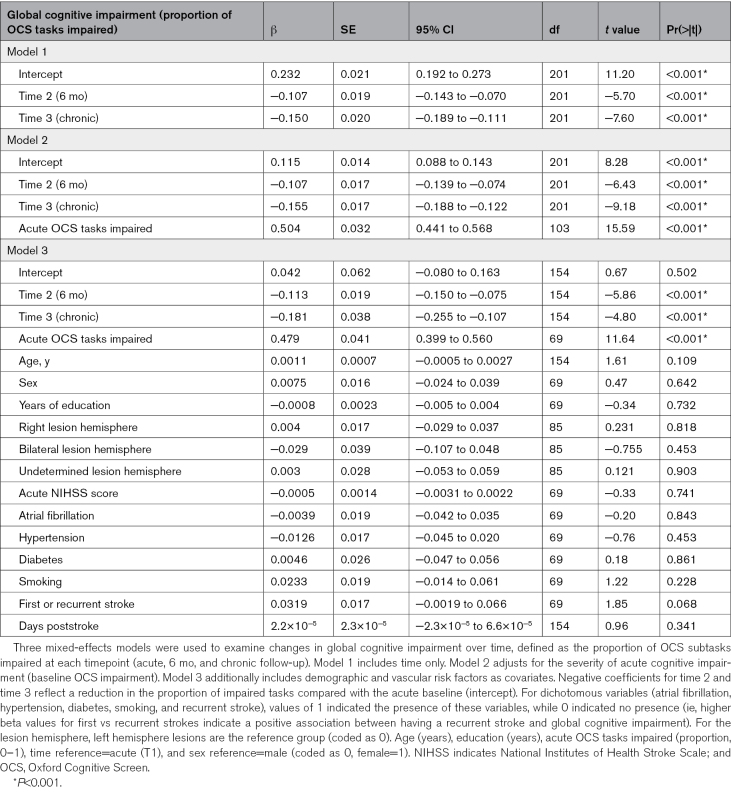
Predicting Changes in Global Cognitive Impairment Over Time (Acute, 6 Months, and Chronic Follow-Up)

### Predicting Domain-Specific Cognitive Impairment Over Time

Mixed-effects logistic regression models (Table S4) showed significant reductions in impairment across all domains. Memory impairment showed the greatest improvement over time, with a high odds of having improved at both postacute follow-up assessments (OR, 3.56 [95% CI, 1.41–8.99] at 6 months and 16.40 [95% CI, 5.52–48.7] at ≥2 years). However, having an acute impairment substantially increased long-term risk (OR, 55.2 [95% CI, 21.5–141.0]; mR^2^=0.60). Similarly, language abilities also improved at 6 months (OR, 3.47 [95% CI, 1.50–8.07]; *P*=0.004) and further at ≥2 years (OR, 8.17 [95% CI, 3.17–21.1]; *P*<0.001), with acute impairment strongly associated with persistent deficits at follow-up (OR, 29.9 [95% CI, 13.3–67.2]; mR^2^=0.51). Attention impairment improved over time (OR, 3.65 [95% CI, 1.81–7.38] at 6 months; OR, 5.41 [95% CI, 2.57–11.4] at ≥2 years), with acute deficits remaining a strong predictor of persistence (OR, 11.1 [95% CI, 5.46–22.6]; mR^2^=0.37). Executive function impairment also improved (OR, 2.86 [95% CI, 1.21–6.77] at 6 months; OR, 4.14 [95% CI, 1.68–10.2] at ≥2 years), with acute impairment linked to a 17-fold increase in long-term risk (OR, 17.9 [95% CI, 8.36–38.40]; mR^2^=0.37). Number processing impairment showed the steepest early improvement (OR, 11.70 [95% CI, 4.35–31.4] at 6 months), which was sustained at follow-up (OR, 7.48 [95% CI, 2.91–19.4]); similarly, acute impairment increased odds of chronic impairment (OR, 33.99 [95% CI, 13.6–84.6]; mR^2^=0.55). Across models, random-effect variance was negligible (ICCadj ≤0.02 or singular), indicating minimal residual between-subject variability after accounting for time and baseline status.

### Trajectories of Global Cognitive Impairment

**Figure 2.**
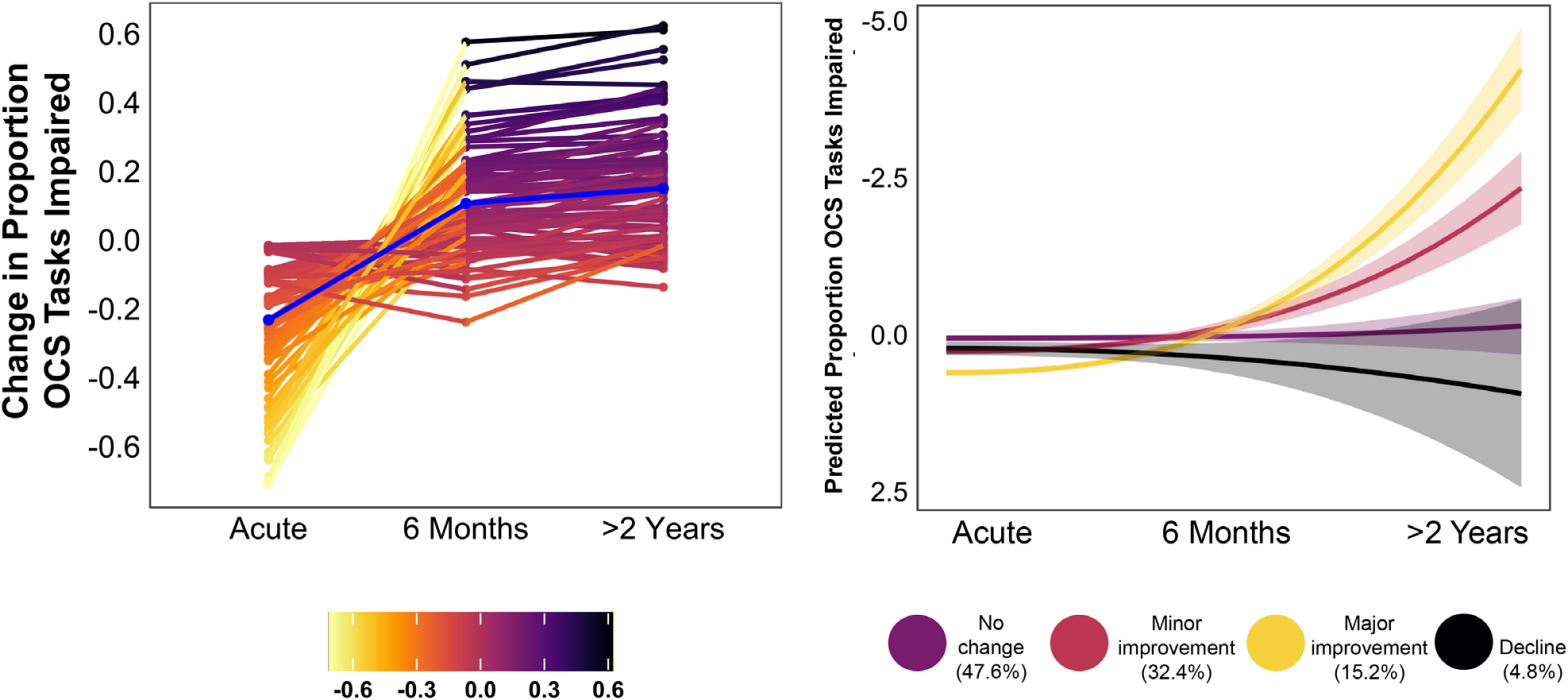
**Trajectories of global cognitive impairment following stroke.** Distinct trajectories of poststroke global cognitive impairment over 3 timepoints (acute, 6 months, and chronic follow-up) for each of the 4 classes identified by Latent Class Growth Modeling. Global cognitive impairment was defined by the proportion of Oxford Cognitive Screen (OCS) tasks impaired. Classes are labeled by color, and the percentage of participants assigned to each latent class is displayed. The left-hand side shows individual change in the raw variable (proportion of OCS tasks impaired) over time, with positive change indicating improvement (in darker colors). The right-hand side shows the predicted proportion of OCS tasks impaired over time per identified latent class.

To examine patient trajectories, we visualized individual-level change in the proportion of OCS tasks impaired from acute hospitalization, 6 months poststroke, and in chronic stroke (Figure 2, left). There appeared to be a global trend of a greater degree of recovery from acute to 6 months; however, there was substantial variability with some patients showing no change or fluctuation across timepoints, further justifying our approach of examining potential trajectory subgroups. In the global cognitive impairment model, the 4-class model demonstrated the best balance between fit, parsimony, and interpretability (AIC=−371.73, BIC=−329.27, SABIC=−379.82, and H=0.80; Table 3). Although the 2-class solution had better entropy (ie, class separation), it was judged to oversimplify the heterogeneity observed in poststroke cognitive recovery profiles. The final model identified 4 distinct trajectories (Figure 2, right).

**Table 3.**
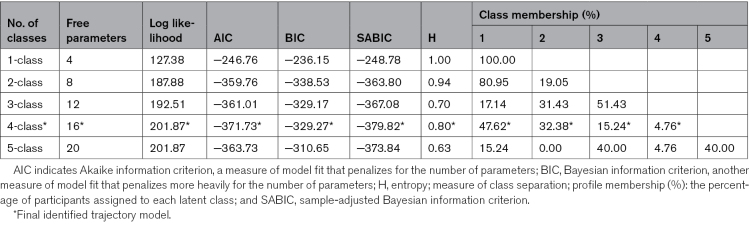
Model Fit Statistics and Class Membership for Latent Class Growth Analysis of Global Cognitive Impairment

1. Class 1: no or mild acute impairment with stable performance over time (47.62%).
2. Class 2: moderate acute impairment with improvement over time (32.38%).
3. Class 3: severe impairment with large improvement over time (15.24%).
4. Class 4: moderate acute impairment with decline (4.76%).

Full model fit statistics and class membership distributions are shown in Table 3. As expected, classes differed on baseline cognitive severity (a greater proportion of OCS tasks impaired) and total number of domains impaired. Chronic Montreal Cognitive Assessment scores also differed across classes (F=12.67; *P*<0.001), with lower scores observed in the declining (M=19.3 [SD, 5.8]) and persistently impaired (M=19.1 [SD, 3.5]) groups compared with those with moderate acute impairment (M=23.3 [SD, 3.1]) and either stable or mild impairment (M=24.8 [SD, 3.5]; Table S1).

### Acute Profiles of Global Cognitive Impairment Subgroups

Full descriptive statistics of acute demographic, clinical, and cognitive characteristics across global cognitive trajectory subgroups are presented in Table S1. Participants with stable mild or no impairments poststroke had more years of education and smaller lesion volumes, and were less likely to show multidomain impairment in the acute phase. The declining subgroup showed the lowest education levels and, along with those with severe acute impairments, showed the lowest Montreal Cognitive Assessment scores at long-term follow-up (M=19.3 [SD, 5.8]).

### Trajectories of Domain-Specific Cognitive Impairments

**Figure 3.**
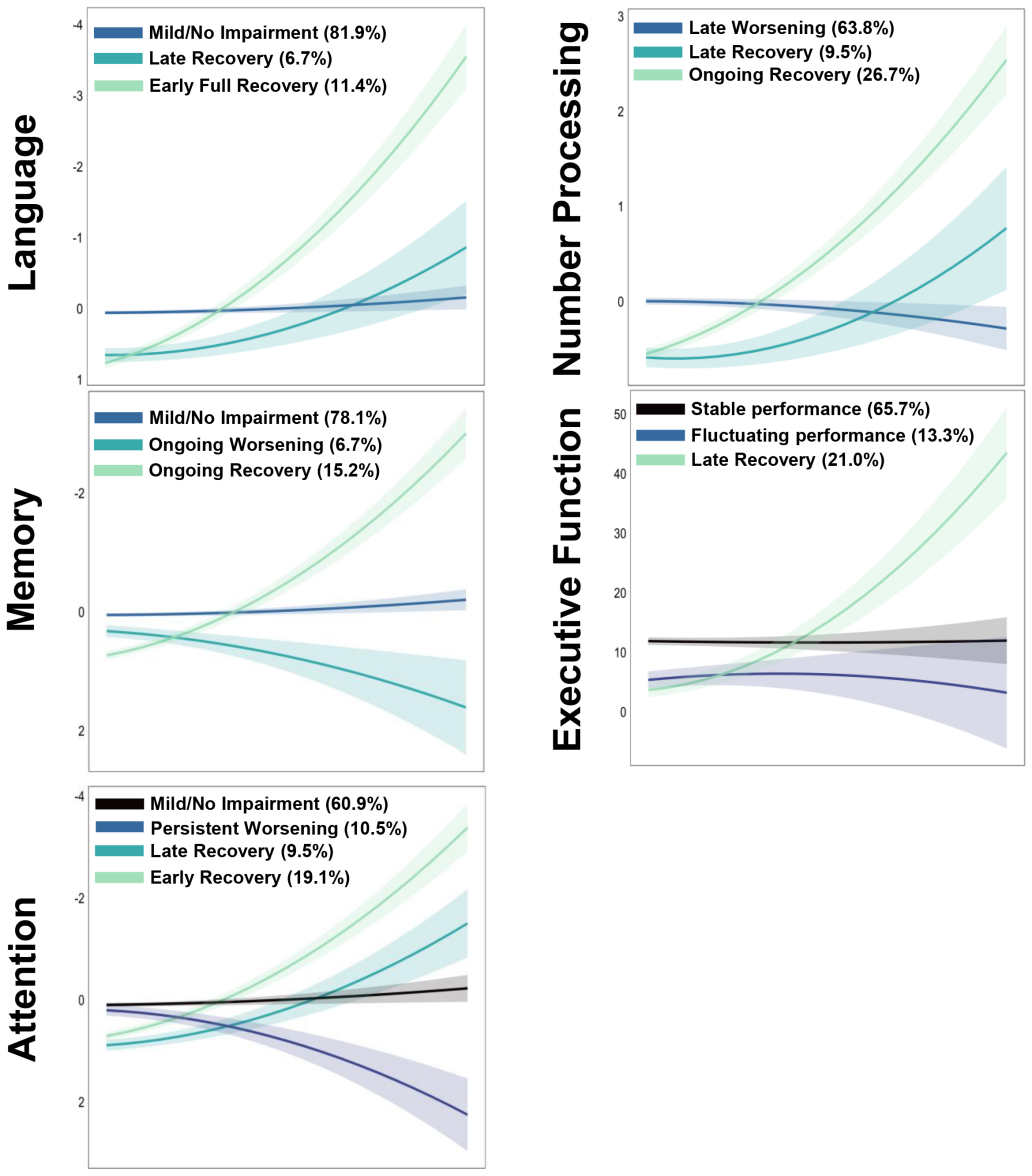
Trajectories of domain-specific cognitive impairments following stroke.

Latent class trajectories for each cognitive domain are visualized in Figure 3, with model fit statistics reported in Tables S5 through S9. A 3-class solution best fit the data for language (AIC=−273.83, BIC=−241.99, SABIC=−279.89, and H=0.99), memory (AIC=−257.75, BIC=−225.90, SABIC=−263.81, and H=0.97), number processing (AIC=−110.98, BIC=−79.13, SABIC=−117.04, and H=0.98), and executive function (AIC=1525.28, BIC=1557.13, SABIC=1519.22, and H=0.90). For attention, a 4-class model provided the best fit (AIC=−29.56, BIC=12.91, SABIC=−37.64, and H=0.92). Across domains, some classes showed similar patterns (eg, late improvements from 6 months to long-term follow-up), while executive function uniquely included a subgroup with fluctuating performance over time. Individual-level raw trajectories for each domain are presented in Figure S2 and illustrate the within-class variability underlying these modeled patterns.

Distinct trajectories of domain-specific cognitive impairment across 3 timepoints (acute, 6 months, and chronic follow-up) are identified using Latent Class Growth Modeling. For the language, memory, attention, and number processing domains, impairment was defined by the proportion of domain-specific subtasks impaired. For executive function, raw scores from the mixed trails subtask were used. Each plot displays predicted class-level trajectories, with class labels and the percentage of participants in each class shown. The *y* axis represents the predicted level of impairment over time.

## Discussion

This study characterized global and domain-specific cognitive trajectories up to 9 years poststroke (mean follow-up, 4.5 years) using repeated, domain-sensitive assessments. In the majority of individuals, both global and domain-specific impairments declined over time, with the greatest recovery occurring within the first 6 months and continued improvement observed in some domains, particularly memory and language. Acute cognitive impairment was the strongest and most consistent predictor of long-term outcomes, while demographic and vascular factors explained minimal additional variance. Importantly, latent class analyses revealed substantial heterogeneity in recovery patterns, including subgroups with delayed improvement or late decline across all domains, highlighting that one-off assessments may fail to capture critical change. These findings underscore the need for individualized monitoring of long-term cognitive outcomes, information provision on possible poststroke trajectories, and customizing cognitive support and rehabilitation based on individual trajectories observed. Future research efforts should aim to replicate trajectories found here and better characterize baseline features using a range of methods so as to better predict recovery trajectories. By identifying replicable markers of trajectories of improvement, persistent impairment, and decline, rehabilitation efforts can be targeted more appropriately.

### Global Cognitive Impairment

A significant reduction in global cognitive impairment over time was observed, with the steepest improvements in function occurring within the first 6 months poststroke and further, though attenuated, gains evident at long-term follow-up (mean, 4.5 [range, 2–9] years). Mixed-effects modeling showed a consistent reduction in the proportion of OCS tasks impaired, going from an average of 3 OCS subtasks impaired at baseline to 1 to 2 OCS subtasks impaired by the 6-month and chronic phase. These findings align with prior studies demonstrating early subacute cognitive recovery^25^ but extend them by showing sustained improvement well beyond the assumed 1-year plateau^26^ and support data from longitudinal cohorts showing recovery up to 2^27^ and even 10 years poststroke.^8,13^ This long-term improvement may reflect a combination of rehabilitation efforts, spontaneous restitution, behavioral compensation, and persistent neuroplasticity.^28,29^ Of note, this may only be true of those assessed here, given that those with worsening impairments withdrew from our sample or passed away.

The strongest and most consistent predictor of global cognitive outcome was the severity of acute cognitive impairment, which accounted for over half the variance in performance across timepoints (R^2^=0.52). However, despite previous meta-analytic findings also supporting a relationship between age, recurrent stroke, and NIHSS scores to poststroke cognition,^17^ we did not observe these relationships in our longitudinal models. Once acute cognitive burden was included, additional demographic, vascular, and clinical variables contributed negligible explanatory power. This mirrors earlier research indicating demographic and clinical variables have weaker relationships to poststroke cognition when compared with acute cognitive status^17^ and domain-level studies showing acute deficits to be the strongest predictors of long-term impairment.^2,9,30^ This suggests that early multidomain cognitive screening may serve as a proxy for global cerebral vulnerability at the time of stroke, incorporating both acute lesion burden and preexisting pathology.^9,31^ Of note is that while acute cognitive status in language, memory, and attention differed by lesion hemisphere, this was not the case at long-term follow-up. This might suggest that such factors more strongly relate to acute cognitive presentation, but variation in access to cognitive rehabilitation, prestroke cognitive and brain reserve, and self-management of symptoms may play a larger role in long-term cognitive impairment. However, it is also possible that demographic factors have comparably small effect sizes relative to acute cognition, only becoming identifiable in meta-analytic work where sample sizes are larger. These findings strongly support the clinical utility of acute cognitive profiling and its inclusion in early stroke care to inform prognostication, discharge planning, and resource allocation, an approach consistent with current guideline recommendations.^7,32^

However, our findings contrast with recent large-scale pooled data from Lo et al,^12^ who reported an acute drop in global cognition followed by an accelerated decline in over 20 000 community-dwelling older adults, with no evidence of poststroke recovery. Several methodological differences may explain this divergence. While Lo et al^12^ harmonized disparate screening tools across cohorts, we used a validated, domain-specific measure (OCS) with high sensitivity to stroke-related cognitive deficits and the ability to distinguish them from sensorimotor symptoms. In addition, our hospital-verified cohort underwent lesion and cognitive phenotyping, and our analytic approach explicitly modeled heterogeneity using latent class growth analysis, rather than assuming a single mean trajectory. Finally, our cohort predominantly included moderate strokes (mean NIHSS score, 7), with a majority showing focal cognitive impairments, whereas the majority of studies on poststroke cognition are biased toward milder stroke samples with lesser opportunity for recovery.

Latent class analysis identified 4 distinct global cognitive recovery patterns. Nearly half the cohort (47.6%) exhibited stable mild or no impairment throughout follow-up. A further 32.4% presented with moderate impairment acutely but improved substantially, and 15.2% showed severe acute deficits with notable long-term gains. In contrast, 4.8% experienced delayed cognitive decline following an acute moderate presentation. This latter class resembles the plateau-then-decline trajectory observed by Delgado et al^33^ and may reflect the impact of progressive small vessel disease, covert recurrent infarction, or emerging neurodegeneration.^31,34^

The clinical implications are considerable. First, these findings support early cognitive screening as a tool for risk stratification and long-term care planning. Second, the substantial recovery seen in individuals with moderate or even severe acute deficits reinforces the potential for ongoing cognitive rehabilitation to maximize improvements made over time. This research also challenges deterministic models of poststroke decline. Finally, the identification of a small but meaningful subgroup with delayed deterioration highlights the need for routine, long-term cognitive monitoring, ideally embedded within a stroke care pathway, to detect emerging decline and intervene appropriately.

### Domain-Specific Cognitive Impairment

Unlike global scores, which may obscure important heterogeneity, domain-level modeling captured differential trajectories that reflect the neuroanatomical and functional specificity of stroke-related damage. Mixed-effects models showed significant reductions in impairment over time across all domains assessed, including language, memory, attention, executive function, and number processing, with the most pronounced recovery typically observed within the first 6 months. However, the degree and persistence of recovery differed by domain.

### Language

Language function showed the most favorable recovery profile across domains. Latent class modeling identified 3 distinct trajectories: the majority (81.9%) exhibited no or only mild impairment throughout follow-up; 11.4% showed early full recovery, while 6.7% showed late improvements. These patterns are consistent with prior studies showing rapid recovery from aphasia within the first 3 months poststroke, often attributed to spontaneous neurobiological restitution.^35^ However, evidence from longitudinal cohorts suggests that meaningful gains can continue well into the chronic phase, likely supported by adaptive functional reorganization and therapy-driven gains.^36^

### Memory

Memory recovery showed a broadly favorable profile, though less consistent than language, with a greater proportion of individuals showing persistent impairment or delayed improvement. Latent class modeling identified 3 trajectories: no or mild acute impairment that remained stable (78.1%), ongoing recovery (15.2%), and ongoing worsening (6.7%). These findings align with previous studies showing early memory gains poststroke, alongside considerable long-term variability.^37,38^ Acute memory impairment remained a strong predictor of long-term outcome, consistent with studies linking early deficits to persistent dysfunction,^39^ though we note that memory was also the most well-recovered domain, potentially linked to language recovery and, therefore, improved verbal encoding. The observed heterogeneity in recovery may reflect the distributed neuroanatomical basis of memory, encompassing medial temporal, prefrontal, and subcortical structures variably affected by stroke and small vessel disease.^37,40^

### Attention

Attentional recovery was more variable than language and memory, with a notable minority of patients exhibiting persistent or worsening deficits. Four trajectory patterns emerged: no or mild impairment across follow-up (60.9%), early recovery (19.1%), delayed recovery (9.5%), and progressive decline (10.5%). These findings confirm substantial heterogeneity often masked by global cognitive scores and align with prior studies showing persistent attention deficits in many stroke survivors, even years after the index event.^41,42^ Persistent impairments in attention are particularly concerning given their association with reduced independence, increased safety risks, and poorer performance across other cognitive domains, including executive function and memory encoding.^2,16^

### Executive Function

Executive function showed the least favorable recovery profile. Three trajectories were identified: stable performance (65.7), fluctuating performance (13.3%), and late recovery (21.0%). Mixed-effects modeling confirmed only a modest reduction in impairment over time (OR, 0.53, ie, a 47% reduction in the odds of full recovery), and acute impairment remained the strongest predictor of long-term outcome. Therefore, a sizeable subgroup experiences executive dysfunction that persists or worsens, with limited spontaneous recovery. This pattern mirrors prior work in both mixed-age and young-stroke cohorts,^43,44^ where only 20% showed meaningful gains at 1 year, while the majority remained stable or declined.^10^ A systematic review further highlights the variability in executive recovery, with many studies failing to demonstrate improvements beyond measurement noise.^6^

Persistent executive dysfunction may reflect the vulnerability of this domain to both focal and diffuse pathology. Executive control relies on distributed frontostriatal and frontoparietal networks, which are particularly sensitive to the effects of white matter disconnection and small vessel disease.^45^ Moreover, persistent executive impairment has been identified as a key predictor of poststroke dementia, particularly in the presence of mixed vascular and neurodegenerative pathology.^16,33^

### Number Processing

Number processing recovery showed considerable heterogeneity. The majority (57.1%) showed stable mild/no impairment, 23.8% showed ongoing improvement, 9.5% showed late improvement, and 9.5% experienced delayed decline. The variability in recovery patterns likely reflects the complex neurocognitive demands of numerical cognition, which rely on distributed frontoparietal and subcortical networks susceptible to both focal and diffuse brain injury.^46,47^

### Strengths and Limitations

This study has several strengths. A key advantage is the long-term, domain-specific cognitive follow-up extending up to 9.3 years poststroke, offering insights into the chronic course of cognitive trajectories. The inclusion of moderate-to-severe stroke survivors and individuals with aphasia, groups often excluded from research,^48^ enhances the generalizability of our findings. Use of the stroke-specific OCS enabled sensitive detection of both overall cognitive impairment severity and domain-level impairments, allowing us to characterize distinct recovery trajectories across cognitive domains. However, various limitations should be acknowledged. Despite best efforts, attrition over time, largely due to death or severe illness, was considerable, as is common in long-term stroke studies, especially in older cohorts. This may have led to underestimation of cognitive impairment and decline, as individuals lost to follow-up likely had more severe deterioration^49^ and cognitive trajectories in more impaired patients; therefore, it remains uncertain. A further limitation is the absence of prestroke cognitive data due to recruitment during acute hospitalization and the lack of data from questionnaires such as the Informant Questionnaire on Cognitive Decline in the Elderly. We were, therefore, unable to determine the impact of any preexisting cognitive impairment on our findings, restricting our ability to disentangle preexisting cognitive decline from stroke effects. Prior studies suggest that individuals who go on to experience stroke may already exhibit accelerated cognitive decline up to a decade before the event.^50^ In addition, one of our cohort inclusion criteria was the ability to concentrate for 20 minutes, which may have skewed the sample toward excluding the more severe stroke phenotypes, though we note our resulting long-term follow-up mainly consisted of moderate stroke. Furthermore, though exploratory analyses between overall cognitive severity subgroups are valuable to guide future prediction of those at risk of decline, some subgroups constituted relatively small numbers of participants, affecting power. In addition, due to the remote nature of follow-up, the praxis domain could not be assessed beyond 6 months, limiting insights into long-term praxis recovery. Finally, while our modeling accounted for a range of clinical and demographic factors, remaining unexplained variance suggests that unmeasured factors, such as rehabilitation intensity, social engagement, or genetic vulnerability, may have contributed to changes in long-term cognition.

Future work should aim to replicate our findings in larger and more diverse samples and, where feasible, incorporate prestroke cognitive measures (eg, through proxy measures of cognition such as the Informant Questionnaire on Cognitive Decline in the Elderly) though these remain challenging to obtain. Longitudinal studies that combine early cognitive phenotyping with detailed neuroimaging may further clarify the mechanisms driving divergent recovery trajectories. In addition, further work is required to enable individualized prediction of specific cognitive trajectories to enable targeted follow-up in those at greatest risk of long-term cognitive decline and to provide reassurance regarding the high likelihood of spontaneous improvement. Finally, examining whether personalized, trajectory-informed interventions improve outcomes relative to standard care represents an important next step for clinical application.

### Conclusions

This study provides rare longitudinal insight into long-term poststroke cognitive recovery, revealing that global and domain-specific functions often improve well beyond the subacute phase, particularly in language, memory, and number processing. Acute cognitive impairment emerged as the strongest and most consistent predictor of long-term outcomes, while demographic and vascular factors added limited explanatory value. Distinct trajectories across all domains, ranging from early and delayed recovery to persistent deficits and late decline, underscore the need to move beyond static models of cognitive outcome. In particular, executive dysfunction and attention deficits were less amenable to spontaneous recovery. These findings highlight the importance of early, domain-sensitive screening to inform prognosis and rehabilitation while supporting a shift toward dynamic, individualized cognitive surveillance. Incorporating trajectory-informed monitoring into stroke care may improve the identification of individuals at risk for decline and guide resource allocation, a strategy that merits prospective evaluation in future trials to determine whether trajectory-based stratification improves functional and cognitive outcomes.

## ARTICLE INFORMATION

### Acknowledgments

The authors thank all participants and members of the Oxford Translational Neuropsychology Group for their contributions to recruitment/testing.

### Disclosures

E. Milosevich reports grants from Alzheimer’s Research UK. Dr Kusec reports grants from the National Institute for Health and Care Research (NIHR). Dr Demeyere is a developer of the Oxford Cognitive Screen, reports grants from the NIHR, reports additional employment by Elsevier Publishing and the University of Edinburgh, and received consultancy payments from Brain Stimulation (SE). S.T. Pendlebury received honoraria from Trondheim, Sydney, and LaTrobe Universities and royalties from Oxford University Press and Cambridge University Press.

### Supplemental Material

Tables S1–S9

Figures S1–S2

STROBE Checklist

## Supplementary Material


