# Supplementary material for: Longitudinal Trajectories of Global and Domain-Specific Cognition After Stroke Using the Oxford Cognitive Screen
Source: Stroke. 2026 May 19;57(7):2150–62. doi: 10.1161/STROKEAHA.125.054555 (PMC13281978; doi:10.1161/STROKEAHA.125.054555)
Supplement: Supplementary file 1 [file str-57-2150-s001.pdf]

SUPPLEMENTAL MATERIAL

Figure S1. Flow-chart of participant recruitment and attrition across all assessment timepoints at acute, 6 months, ≥2 years post-stroke (chronic).

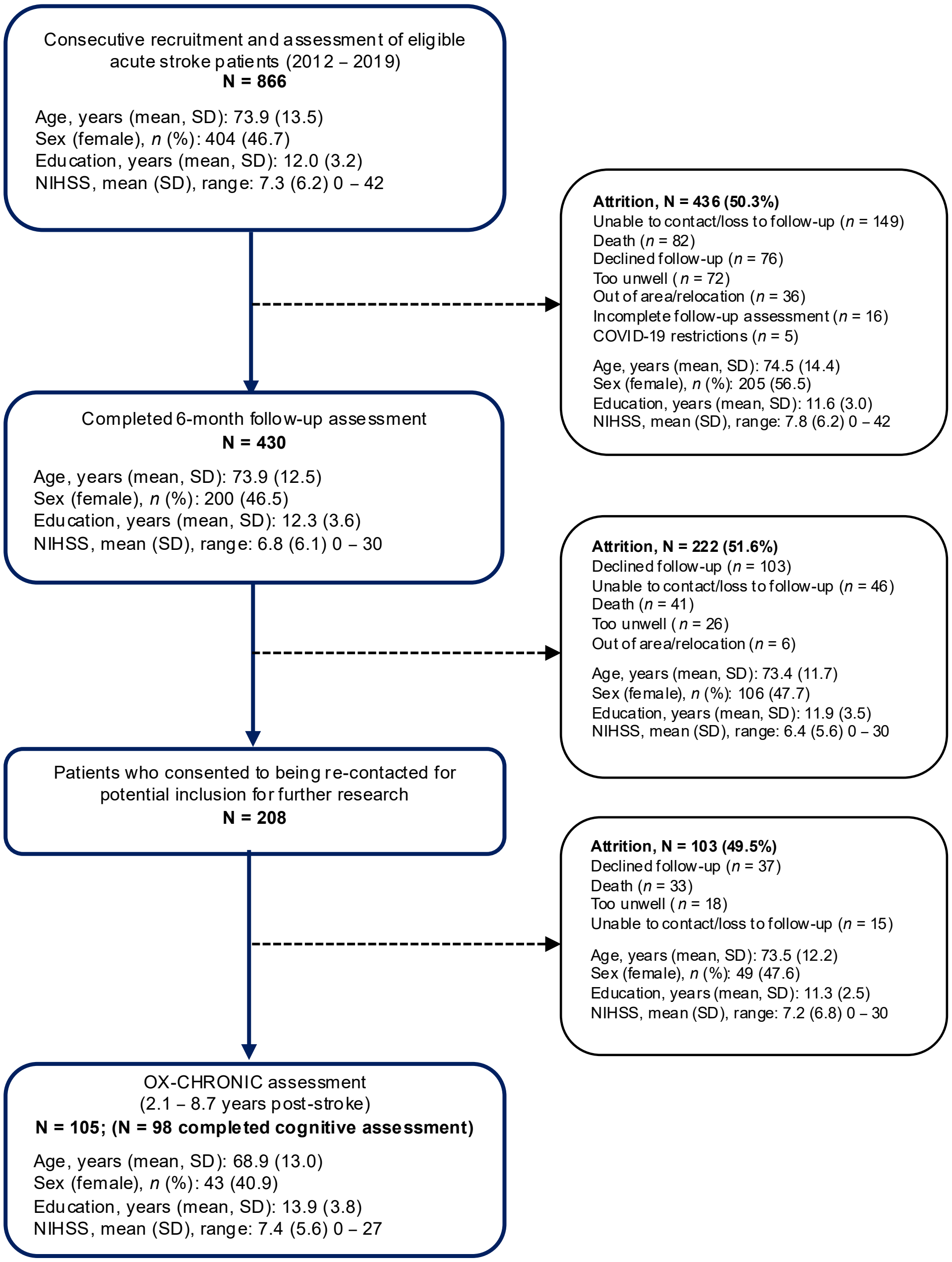

Table S1. Demographic, clinical, and cognitive characteristics of global cognitive trajectory subgroups.

	**Full Sample** (n = 105)	**Group 1** Stable Mild/No Impairment (n = 50)	**Group 2** Moderate Acute Impairment with Improvement (n = 34)	**Group 3** Severe Acute Impairment with Improvement (n = 16)	**Group 4** Decliners (n = 5)	Omnibus Test statistic (*p-value*)
Age	68.9 (13.0)	70.2 (13.2)	69.4 (12.9)	63.4 (13.1)	69.2 (11.2)	*F* = 1.15 (0.33)
Sex	59.0% Male	56.0% Male	64.7% Male	56.3% Male	60.0% Male	X2 = 0.69 (0.87)
NIHSS	7.4 (5.6)	6.8 (5.9)	6.9 (4.2)	10.9 (6.9)	5.4 (2.3)	***F* = 2.78 (<0.05)**
Lesion Hemisphere	40.0% Right	36.0% Right	47.1% Right	31.3% Right	40.0% Right	X2 = 12.54 (0.18)
Years of Education	13.9 (3.6)	15.5 (3.8)	12.6 (2.8)	12.9 (3.4)	11.2 (2.2)	***F* = 6.57 (<0.001)**
Stroke Type	83.8% Ischaemic	84.0% Ischaemic	88.2% Ischaemic	75.0% Ischaemic	80.0% Ischaemic	X2 = 1.46 (0.69)
First vs Recurrent	66.7% First	68.0% First	70.6% First	62.5% First	40.0% First	X2 = 2.00 (0.57)
Lesion Volume (mm^3)	34142.2 (39364.8)	22201.7 (23590.8)	37550.1 (64130.6)	64130.6 (63869.0)	34410.4 (46123.4)	***F* = 5.29 (<0.01)**
Acute Prop Impaired	0.23 (0.2)	0.06 (0.07)	0.30 (0.08)	0.62 (0.10)	0.23 (0.15)	***F* = 186.82 (<0.001)**
Domains Impaired	57.1% 2+ Domains	16.0% 2+ Domains	97.1% 2+ Domains	100.0% 2+ Domains	60.0% 2+ Domains	***X2* = 135.39 (<0.001)**
GCA Total	10.4 (7.2)	9.3 (7.5)	10.4 (6.5)	11.7 (6.3)	11.2 (4.9)	*F* = 2.19 (0.09)
Fazekas Total	1.5 (1.5)	1.2 (1.3)	1.5 (1.5)	1.9 (1.7)	2.6 (2.3)	*F* = 2.03 (0.12)
Time 3 MoCA	23.4 (4.2)	24.8 (3.5)	23.3 (3.1)	19.1 (3.5)	19.3 (5.8)	***F* = 12.67 (<0.001)**

Descriptive statistics of the full study sample and identified subgroups via latent class growth analysis. Test statistics represent any identified differences between subgroups. NIHSS=National Institute of Health Stroke Severity; Acute Prop Impaired = Acute Proportion of Oxford Cognitive Screen Tasks Impaired; GCA=Global Cortical Atrophy scale total; MoCA=Montreal Cognitive Assessment

Table S2. Point prevalence of domain-specific cognitive impairments at chronic follow-up (n=98).

**OCS impairment**	2 – 9.4 years post-stroke (n=98) **N (%)**	2 – 5 years post-stroke (n=66) **N (%)**	≥5 years post-stroke (n=32) **N (%)**
**Language**	11 (11.22)	5 (7.58)	6 (18.75)
Picture naming	4 (4.08)	1 (1.52)	3 (9.38)
Semantic understanding	0 (0.00)	0 (0.00)	0 (0.00)
Sentence reading	7 (7.14)	4 (6.06)	3 (9.38)
**Attention**	14 (14.29)	11 (16.67)	3 (9.38)
Egocentric attention	8 (8.16)	7 (10.61)	1 (3.13)
Allocentric attention	8 (8.16)	6 (9.09)	2 (6.25)
**Executive Function**	12 (12.24)	6 (9.09)	6 (18.75)
**Memory**	8 (8.16)	5 (7.58)	3 (9.38)
Orientation	4 (4.08)	2 (3.03)	2 (6.25)
Verbal memory	5 (5.10)	4 (6.06)	1 (3.13)
Episodic memory	1 (1.02)	1 (1.52)	0 (0.00)
**Number processing**	13 (13.27)	8 (12.12)	5 (15.63)
Calculations	3 (3.06)	1 (1.52)	2 (6.25)
Writing	12 (12.24)	8 (12.12)	4 (12.50)
**Any domain**	41 (41.84)	26 (39.39)	15 (46.88)
**Single domain**	30 (30.61)	19 (28.79)	11 (24.38)
**Multi-domain**	11 (11.22)	7 (10.61)	4 (12.50)
MoCA (mean, SD)	23.14 (4.21)	23.32 (4.23)	22.78 (4.21)
MoCA <26	64 (65.31)	41 (62.12)	23 (71.88)

Prevalence of impairment within each domain at long-term follow-up. Any domain impairment was defined by an impairment on any subtest in any domain; multi-domain refers to impairment in more than one domain. The second column refers to those who were between 2-5 years post-stroke at long-term follow-up (Time 3) and the third column refers to those who were greater than 5 years post-stroke at long-term follow-up. OCS: Oxford Cognitive Screen; MoCA: Montreal Cognitive Assessment.

Table S3. Stratification of acute (<2 weeks post-stroke) and chronic (>2 years) domain impairment by lesion hemisphere

Acute Cognitive Status

	Overall PSCI		Language		Attention		Executive Function		Memory		Number Processing	
	Not impaired (*n*=23)	Impaired (*n*=82)	Not impaired (*n*=69)	Impaired (*n*=36)	Not impaired (*n*=45)	Impaired (*n*=55)	Not impaired (*n*=75)	Impaired (*n*=25)	Not impaired (*n*=69)	Impaired (*n*=36)	Not impaired (*n*=68)	Impaired (*n*=37)
**Left Hemisphere**	34.8%	41.5%	30.4%	58.3%	44.4%	36.4%	44.0%	32.0%	28.9%	61.1%	35.3%	48.6%
**Right Hemisphere**	34.8%	40.2%	42.0%	33.3%	22.2%	50.9%	32.0%	60.0%	46.4%	25.0%	36.8%	43.2%
**Bilateral**	8.7%	7.3%	11.6%	0.0%	8.9%	7.3%	9.3%	4.0%	8.7%	5.6%	8.8%	5.4%
**Undetermined from Scan**	21.7%	10.9%	15.9%	8.3%	24.4%	5.5%	14.7%	4.0%	15.9%	8.3%	19.1%	2.7%
**Omnibus Test Statistic**	χ^2=1.9		χ^2=10.3*		χ^2=12.2**		χ^2=6.9		χ^2=10.2*		χ^2=6.5	

Chronic Cognitive Status

	Overall PSCI		Language		Attention		Executive Function		Memory		Number Processing	
	Not impaired (*n*=52)	Impaired (*n*=46)	Not impaired (*n*=87)	Impaired (*n*=11)	Not impaired (*n*=73)	Impaired (*n*=25)	Not impaired (*n*=86)	Impaired (*n*=12)	Not impaired (*n*=90)	Impaired (*n*=8)	Not impaired (*n*=68)	Impaired (*n*=37)
**Left Hemisphere**	40.4%	41.3%	41.4%	36.4%	41.1%	40.0%	41.9%	33.3%	40.0%	50.0%	38.8%	53.8%
**Right Hemisphere**	34.6%	45.6%	37.9%	54.5%	35.6%	52.0%	38.4%	50.0%	40.0%	37.5%	40.0%	38.5%
**Bilateral**	9.6%	4.3%	6.9%	9.1%	8.2%	4.0%	8.1%	0.0%	7.8%	0.0%	8.2%	0.0%
**Undetermined from Scan**	15.4%	8.7%	13.8%	0.0%	15.1%	4.0%	11.6%	16.7%	12.2%	12.5%	12.9%	7.7%
**Omnibus Test Statistic**	χ^2=2.6		χ^2=2.3		χ^2=3.6		χ^2=1.7		χ^2=0.81		χ^2=1.9	

Percentage of participants with left, right, bilateral or undetermined lesion hemisphere stratified by global and domain-specific impairment status at time of acute stroke and in chronic stroke (>2 years) as measured by the Oxford Cognitive Screen. PSCI=post-stroke cognitive impairment. Significance levels: ***$p < 0.001$, **$p < 0.01$, *$p < 0.05$.

Table S4. Predicting changes in domain-specific cognitive impairments over time (acute, 6 months, chronic/long-term follow-up).

Domain-specific impairment	Log odds	*OR*	*SE*	95% CI	z-value	Pr(>|z|)
**Language**						
(Intercept)	-1.314	0.27	0.355	[0.13, 0.54]	-3.701	<0.001 ***
Time 2 (6 Months)	1.245	3.47	0.430	[1.50, 8.07]	2.896	0.004 **
Time 3 (Chronic)	2.101	8.17	0.483	[3.17, 21.1]	4.346	<0.001 ***
Acute Language Impaired	3.399	29.9	0.413	[13.3, 67.2]	-8.237	<0.001 ***
Language: mR² = 0.506, ICC_{adj} = 0.00						
**Memory**						
(Intercept)	-1.635	0.20	0.400	[0.09, 0.43]	-4.088	<0.001 ***
Time 2 (6 Months)	1.271	3.56	0.472	[1.41, 8.99]	2.693	0.007 **
Time 3 (Chronic)	2.797	16.40	0.555	[5.52, 48.7]	5.038	<0.001 ***
Acute Memory Impaired	4.010	55.20	0.480	[21.5, 141.0]	8.359	<0.001 ***
Memory: mR² = 0.601, ICC_{adj} = 0.00						
**Attention**						
(Intercept)	-1.336	0.26	0.299	[0.15, 0.47]	–4.465	<0.001 ***
Time 2 (6 Months)	1.295	3.65	0.359	[1.81, 7.38]	3.607	<0.001 ***
Time 3 (Chronic)	1.688	5.41	0.381	[2.57, 11.4]	4.434	<0.001 ***
Acute Attention Impaired	2.409	11.10	0.363	[5.46, 22.6]	6.644	<0.001 ***
Attention: mR² = 0.369, ICC_{adj}= 0.02						
**Executive function**						
(Intercept)	-0.783	0.46	0.355	[0.23, 0.92]	-2.205	0.027 *
Time 2 (6 Months)	1.051	2.86	0.439	[1.21, 6.77]	2.389	0.017 *
Time 3 (Chronic)	1.421	4.14	0.462	[1.68, 10.2]	3.078	0.002 **
Acute Executive Impaired	2.886	17.90	0.389	[8.36, 38.40]	7.420	<0.001 ***
Executive function: mR² = 0.377, ICC_{adj} = 0						
**Number processing**						
(Intercept)	-1.404	0.25	0.360	[0.12, 0.51]	–3.736	<0.001 ***
Time 2 (6 Months)	2.457	11.7	0.504	[4.35, 31.4]	4.876	<0.001 ***
Time 3 (Chronic)	2.012	7.48	0.482	[2.91, 19.4]	4.176	<0.001 ***
Acute Number Impaired	3.523	33.99	0.467	[13.6, 84.6]	7.548	<0.001 ***
Number processing: mR² = 0.550, ICC_{adj} = 0						

Mixed-effects logistic regression models for each cognitive domain. The table shows log odds, odds ratios (OR), standard errors (SE), 95% confidence intervals (CI), z-values, and p-values for the fixed effects. Model performance metrics include marginal R^2 (mR^2) and adjusted intraclass correlation coefficient (ICCadj). In models where singular fits were detected (i.e., random effect variance ≈ 0), ICCadj is not reported. Significance levels: ***$p < 0.001$, **$p < 0.01$, *$p < 0.05$.

LANGUAGE DOMAIN

Table S5. Model fit statistics and class membership for Latent Class Growth Analysis of Language Impairment.

**Number of classes**	Free parameters	Log Likelihood	AIC	BIC	SABIC	Entropy	Profile Membership (%)				
							1	2	3	4	5
1-class	4	32.29	-56.57	-45.96	-58.59	1.00	100.00				
2-class	8	107.01	-198.02	-176.79	-202.06	0.95	81.9	18.1			
**3-class**	**12**	**148.92**	**-273.83**	**-241.99**	**-279.89**	**0.99**	**6.7**	**81.9**	**11.4**		
4-class	16	170.83	-306.44	-263.97	-314.52	0.98	12.4	70.5	5.7	11.4	
5-class	20	201.46	-362.92	-309.84	-373.02	0.99	5.7	11.4	12.4	59.1	11.4

AIC: The Akaike Information Criterion, a measure of model fit that penalizes for the number of parameters; BIC: The Bayesian Information Criterion, another measure of model fit that penalizes more heavily for the number of parameters; SABIC: Sample-Adjusted BIC; Entropy: Measure of class separation; Profile Membership (%): The percentage of participants assigned to each latent class.

MEMORY DOMAIN

Table S6. Model fit statistics and class membership for Latent Class Growth Analysis of Memory Impairment.

Number of classes	Free parameters	Log Likelihood	AIC	BIC	SABIC	Entropy	Profile Membership (%)				
							1	2	3	4	5
1-class	4	43.56	-79.11	-68.49	-81.13	1.00	100.0				
2-class	8	107.88	-199.75	-178.52	-203.79	0.92	80.0	20.0			
**3-class**	**12**	**140.88**	**-257.75**	**-225.90**	**-263.81**	**0.97**	**6.7**	**78.1**	**15.2**		
4-class	16	166.52	-301.05	-258.58	-309.13	0.97	63.8	19.0	6.7	10.5	
5-class	20	201.88	-363.76	-310.68	-373.86	0.98	9.5	17.1	9.5	5.7	58.1

AIC: The Akaike Information Criterion, a measure of model fit that penalizes for the number of parameters; BIC: The Bayesian Information Criterion, another measure of model fit that penalizes more heavily for the number of parameters; SABIC: Sample-Adjusted BIC; Entropy: Measure of class separation; Profile Membership (%): The percentage of participants assigned to each latent class.

ATTENTION DOMAIN

Table S7. Model fit statistics and class membership for Latent Class Growth Analysis of Attention Impairment.

							Profile Membership (%)				
**Number of classes**	Free parameters	Log Likelihood	AIC	BIC	SABIC	Entropy	1	2	3	4	5
1-class	4	-47.23	102.46	113.08	100.44	1.00	100.0				
2-class	8	-18.83	53.66	74.89	49.62	0.78	19.1	80.9			
3-class	12	9.33	5.34	37.19	-0.72	0.88	65.7	19.1	15.2		
**4-class**	**16**	**30.78**	**-29.56**	**12.91**	**-37.64**	**0.92**	**9.5**	**19.1**	**60.9**	**10.5**	
5-class	20	32.55	-23.02	27.97	-35.21	0.83	11.4	7.6	19.1	42.9	19.1

AIC: The Akaike Information Criterion, a measure of model fit that penalizes for the number of parameters; BIC: The Bayesian Information Criterion, another measure of model fit that penalizes more heavily for the number of parameters; SABIC: Sample-Adjusted BIC; Entropy: Measure of class separation; Profile Membership (%): The percentage of participants assigned to each latent class.

EXECUTIVE FUNCTION DOMAIN

Table S8. Model fit statistics and class membership for Latent Class Growth Analysis of Executive Function (Trails Total).

**Number of classes**	Free parameters	Log Likelihood	AIC	BIC	SABIC	Entropy	1	2	3	4
1-class	4	-815.34	1638.68	1649.29	1636.66	1.00	100.00			
2-class	8	-780.63	1577.25	1598.49	1573.21	0.81	66.67	33.3		
**3-class**	**12**	**-750.63**	**1525.28**	**1557.13**	**1519.22**	**0.90**	**65.7**	**13.3**	**21.0**	
4-class	16	-750.64	1533.28	1575.75	1525.19	0.69	0.00	64.76	14.29	20.95

AIC: The Akaike Information Criterion, a measure of model fit that penalizes for the number of parameters; BIC: The Bayesian Information Criterion, another measure of model fit that penalizes more heavily for the number of parameters; SABIC: Sample-Adjusted BIC; Entropy: Measure of class separation; Profile Membership (%): The percentage of participants assigned to each latent class.

NUMBER PROCESSING DOMAIN

Table S9. Model fit statistics and class membership for Latent Class Growth Analysis of Number Processing.

**Number of classes**	Free parameters	Log Likelihood	AIC	BIC	SABIC	Entropy	1	2	3	4	5
1-class	4	-4.86	17.71	28.33	15.69	1.00	100.00				
2-class	8	24.57	-33.13	-11.89	-37.17	0.95	12.4	87.6			
**3-class**	**12**	**67.49**	**-110.98**	**-79.13**	**-117.04**	**0.98**	**63.8**	**9.5**	**26.7**		
4-class	16	124.16	-216.32	-173.85	-224.39	0.99	9.5	57.1	23.8	9.5	
5-class	20	124.16	-208.32	-155.24	-218.42	0.75	0.00	57.1	9.5	9.5	23.8

AIC: The Akaike Information Criterion, a measure of model fit that penalizes for the number of parameters; BIC: The Bayesian Information Criterion, another measure of model fit that penalizes more heavily for the number of parameters; SABIC: Sample-Adjusted BIC; Entropy: Measure of class separation; Profile Membership (%): The percentage of participants assigned to each latent class.

Figure S2. Individual-level raw trajectories.

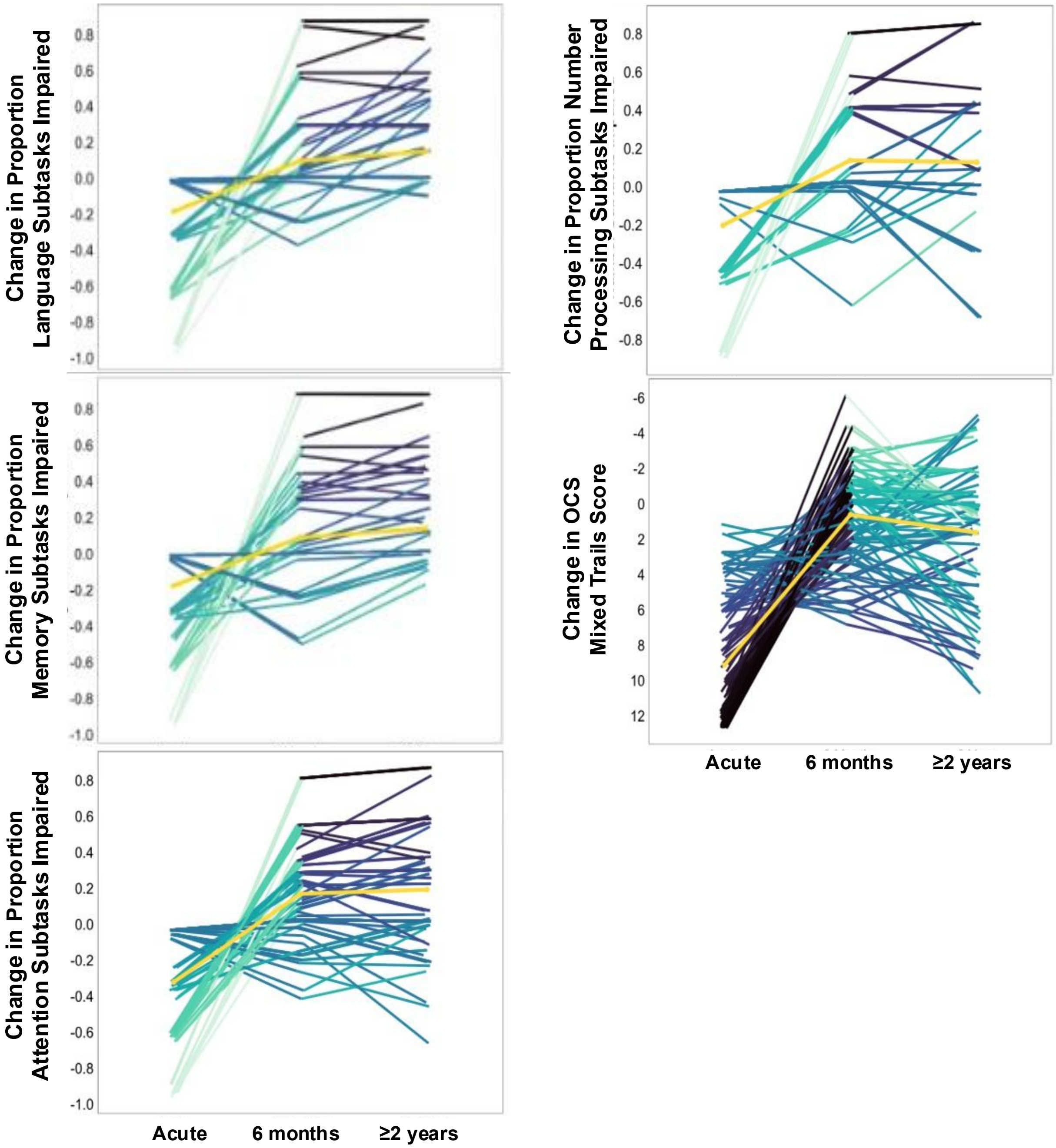

Individual-level trajectories of domain-specific cognitive impairment based on raw data. Each line represents a participant's impairment level across the three timepoints (acute, 6 months, chronic [≥2 years] follow-up). Lines are jittered slightly to represent where there are overlapping individual trajectories. This visualization complements the model-based trajectories shown in Figure 3 and highlights within-individual variability.
